# Case Report: A germline *SDHB* c.268C>T (p.Arg90Ter) variant in a family with very-early-onset colorectal neoplasia

**DOI:** 10.3389/fonc.2026.1938314

**Published:** 2026-10-16

**Authors:** Chang Liu, Dayang Zhou, Wenjing Xu, Hai Pan, Xiang Ji, Jian Huang, Zhu Zhu

**Affiliations:** 1 Day Surgery Center, The First Affiliated Hospital of Kunming Medical University, Kunming, China; 2 Surgical Oncology, The First Affiliated Hospital of Kunming Medical University, Kunming, China

**Keywords:** case report, colorectal cancer, familial colorectal neoplasia, germline variant, *SDHB*, *TP53*, very-early-onset colorectal cancer

## Abstract

**Background:**

Very-early-onset colorectal cancer (CRC), particularly when clustered within a family, raises concern for an inherited cancer predisposition. However, the contribution of germline *SDHB* variants to CRC susceptibility remains uncertain. We report a family in which two sisters developed colorectal carcinoma before 25 years of age and in whom a known pathogenic germline *SDHB* variant was identified.

**Case presentation:**

A 22-year-old woman presented with a 3-year history of intermittent hematochezia. Complete colonoscopy identified an ulcerated lesion in the rectosigmoid region approximately 15 cm from the anal verge. Endoscopic biopsy showed high-grade intraepithelial neoplasia with focal intramucosal carcinoma. The patient underwent laparoscopic rectal resection with lymph-node dissection and sigmoidorectal anastomosis. The surgical resection specimen demonstrated moderately differentiated adenocarcinoma, pT3N0M0, stage IIA, with no metastatic involvement in 14 examined lymph nodes. Germline testing identified *SDHB* NM_003000.3:c.268C>T (p.Arg90Ter). Her elder sister had developed rectal adenocarcinoma at 19 years of age, whereas their father had two rectal polyps measuring approximately 0.6 and 0.8 cm, with histopathology demonstrating tubular adenoma. The same germline *SDHB* variant was detected in the proband, her elder sister, and her father. In the proband’s tumor, the *SDHB* variant showed a VAF of 20.75%, SDHB protein expression was retained, and no biallelic *SDHB* inactivation was demonstrated. The tumor additionally harbored a somatic *TP53* frameshift alteration and *SMAD4* copy-number loss.

**Conclusions:**

This family demonstrates remarkable clustering of very-early-onset colorectal neoplasia together with a known pathogenic germline *SDHB* variant. However, the available segregation, molecular, and immunohistochemical evidence is insufficient to establish *SDHB* as a CRC susceptibility gene or to demonstrate an *SDHB*-driven tumor mechanism. This observation should therefore be considered hypothesis-generating and warrants validation in additional families, larger cohorts, comprehensive genomic analyses, and CRC-specific functional studies.

## 1 Introduction

Colorectal cancer (CRC) remains one of the most commonly diagnosed malignancies worldwide and is a leading cause of cancer-related mortality (1). Very-early-onset CRC is of particular concern for inherited cancer predisposition, and multigene germline testing has identified pathogenic cancer-susceptibility variants in a clinically meaningful proportion of patients with early-onset CRC (2, 3). With the rapid development of molecular biology, numerous genetic factors driving CRC have been identified. *SDHB* encodes the iron-sulfur subunit of mitochondrial complex II and participates in both the tricarboxylic acid cycle and the electron-transport chain. Pathogenic germline *SDHB* variants are established causes of hereditary tumor predisposition, particularly pheochromocytoma/paraganglioma, and have also been associated with other recognized SDH-related tumors, including gastrointestinal stromal tumor and renal cell carcinoma (4). In contrast, colorectal carcinoma is not currently an established component of the classical *SDHB*-associated tumor spectrum. The *SDHB* c.268C>T (p.Arg90Ter) variant identified in the present family is a known pathogenic loss-of-function variant in established *SDHB*-related disorders (5, 6). However, its disease-specific relevance to colorectal cancer remains uncertain. In the present family, two sisters developed colorectal carcinoma at 19 and 22 years of age, respectively, and both carried the *SDHB* variant. Their father also carried the variant and had rectal tubular adenoma. This unusual familial clustering raises the possibility of an underlying hereditary predisposition but does not establish *SDHB* as the responsible colorectal cancer susceptibility gene. We therefore present this family as a hypothesis-generating clinical observation and examine the potential significance and limitations of the genetic findings.

## 2 Case presentation

A 22-year-old woman was admitted in June 2022 with a 3-year history of intermittent hematochezia and recent worsening of abdominal symptoms. Complete colonoscopy performed on June 10, 2022 was advanced to the cecum and approximately 8 cm into the terminal ileum. No significant mucosal abnormalities were identified in the terminal ileum, cecum, ascending colon, transverse colon, or descending colon. An ulcerated lesion involving approximately half of the luminal circumference was identified approximately 15 cm from the anal verge (**Figure 1A**). The original endoscopy report described the lesion as being located in the distal sigmoid colon. Contact bleeding was noted, and biopsy specimens were obtained. Internal hemorrhoids were also documented. Histopathological examination of the endoscopic biopsy showed high-grade intraepithelial neoplasia with focal intramucosal carcinoma. The clinical timeline and major milestones of the diagnostic and treatment process are summarized in **Table 1**.

**Figure 1:**
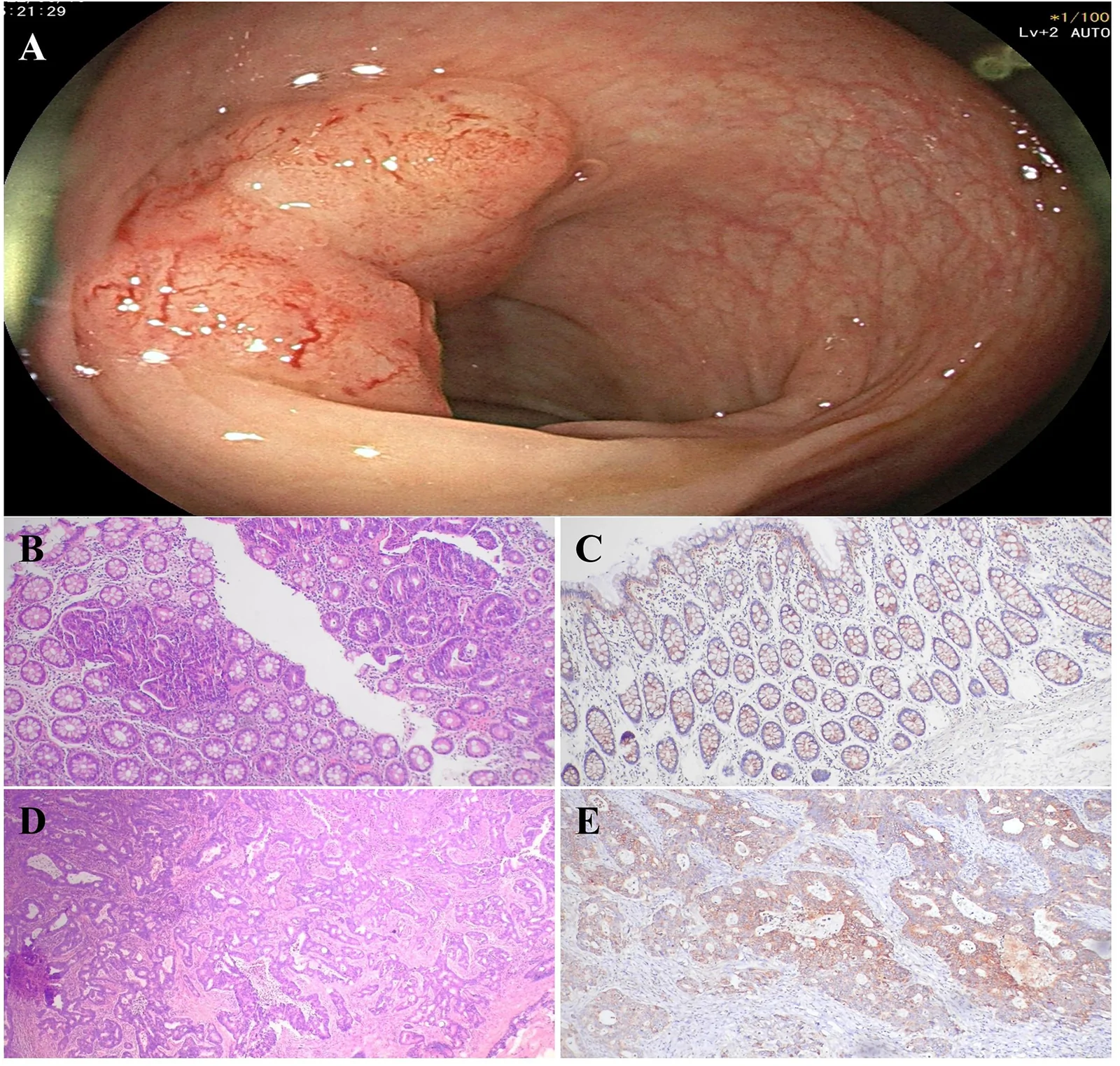
Endoscopic, histopathological, and immunohistochemical findings in the proband. **(A)** Colonoscopic view of the rectosigmoid lesion approximately 15 cm from the anal verge. **(B)** Hematoxylin and eosin staining of adjacent colonic mucosa and the transition zone. **(C)** SDHB immunohistochemistry in adjacent normal mucosa showing granular cytoplasmic expression and serving as an internal positive control. **(D)** Hematoxylin and eosin staining of the tumor showing moderately differentiated adenocarcinoma. **(E)** SDHB immunohistochemistry showing retained granular cytoplasmic expression in the adenocarcinoma. Retained SDHB expression does not establish normal enzymatic activity but provides no immunohistochemical evidence of a conventional SDH-deficient tumor phenotype.

**Table 1:** Clinical timeline of the proband.

Time	Clinical event
3 years before diagnosis	Onset of intermittent hematochezia and fecal urgency
June 2022	Worsening hematochezia and abdominal symptoms; hospital evaluation
June 10, 2022	Complete colonoscopy identified a rectosigmoid lesion approximately 15 cm from the anal verge
June 11, 2022	Endoscopic biopsy showed high-grade intraepithelial neoplasia with focal intramucosal carcinoma
June 15, 2022	Laparoscopic rectal resection with lymph-node dissection and sigmoidorectal anastomosis
Postoperative pathology (June 2022)	Moderately differentiated adenocarcinoma, pT3N0M0, stage IIA; 0/14 lymph nodes
August 2022	Follow-up colonoscopy showed postoperative changes at the anastomotic site without another significant colorectal lesion
February 2023	Follow-up CT showed no evident abnormal wall thickening or abnormal enhancement around the anastomosis

At admission, no abnormalities were found in the physical examination, including body temperature 36.5 °C, pulse rate 80 beats/min, respiratory rate 18 beats/min, and blood pressure 110/74 mmHg. Serum tumor marker evaluation revealed carcinoembryonic antigen (CEA) levels of 4.21 ng/mL (normal range: <5 ng/mL) and carbohydrate antigen 19-9 (CA19-9) levels <0.6 U/mL (normal range: <30 U/mL). On June 15, 2022, the patient underwent laparoscopic rectal resection with lymph-node dissection and sigmoidorectal anastomosis under general anesthesia. Histopathological examination of the surgical resection specimen demonstrated a moderately differentiated adenocarcinoma measuring approximately 1.7 × 1.0 × 0.7 cm. The tumor invaded through the bowel wall but did not penetrate the serosal surface. No lymphovascular or perineural invasion was identified, the resection margins were negative, and none of the 14 examined lymph nodes contained metastatic carcinoma. The postoperative record classified the tumor as pT3N0M0, stage IIA. Immunohistochemical examination showed retained expression of MLH1, MSH2, MSH6, and PMS2. p53 immunostaining was reported as negative. The patient recovered after surgery and was subsequently discharged.

Surgically resected tumor tissue and peripheral blood from the proband were subjected to next-generation sequencing. Germline analysis was performed using an 87-gene hereditary cancer panel, with the complete gene list provided in the **Supplementary Methods** Genetic Analysis. The *SDHB* variant is reported according to the MANE Select transcript as NM_003000.3:c.268C>T (p.Arg90Ter). Key genetic annotation of the germline variant is summarized in **Table 2**. Population-frequency assessment in the original clinical sequencing workflow included the 1000 Genomes Project and ExAC databases, and variant pathogenicity was interpreted according to ACMG/AMP criteria (7). The exact numerical minor-allele-frequency threshold and the complete set of in-silico prediction algorithms could not be reliably reconstructed from the currently available laboratory documentation and therefore were not retrospectively inferred or used as independent evidence in the present study. Genomic profiling identified the heterozygous germline *SDHB* NM_003000.3:c.268C>T (p.Arg90Ter) variant. Somatic analysis of the tumor identified a *TP53* frameshift alteration (p.L93Pfs*56) with a VAF of 57.66% and *SMAD4* copy-number loss. The *SDHB* variant showed a VAF of 20.75% in the tumor specimen. No biallelic *SDHB* inactivation was demonstrated by the available analysis. Because variant allele frequency alone cannot establish allele-specific copy-number status, no inference regarding retention or loss of the wild-type allele was made solely from the VAF. SDHB immunohistochemistry showed retained, strong granular cytoplasmic staining in tumor cells, with adjacent normal colonic mucosa serving as an internal positive control. Although retained SDHB expression does not establish normal enzymatic activity, it provides no immunohistochemical evidence of a conventional SDH-deficient tumor phenotype (**Figures 1B–E**).

**Table 2:** Genetic annotation of the germline *SDHB* c.268C>T (p.Arg90Ter) variant.

Item	Reported information
Gene	*SDHB*
Reference transcript	NM_003000.3
Reference genome	GRCh37/hg19
Genomic variant	NC_000001.10:g.17359573G>A
Coding variant	c.268C>T
Protein consequence	p.Arg90Ter
Zygosity	Heterozygous
Established disease context	Pathogenic in established SDHB-related disorders
Relevance to colorectal cancer	Unconfirmed

The tumor was microsatellite stable (MSS), and the laboratory-reported tumor mutational burden was 10.72 mutations/Mb. According to the original laboratory report, this value corresponded to the 90.41st percentile of the laboratory’s historical tumor dataset and was classified as “high” using its internal percentile-based reference system. This designation was assay- and laboratory-specific and was not interpreted as a universally validated CRC-specific threshold. Common colorectal cancer driver genes, including *KRAS, NRAS, and BRAF*, were wild type in the tumor. No pathogenic germline variants were identified in the tested mismatch-repair and polyposis-associated genes, including *MLH1, MSH2, MSH6, PMS2, APC, and MUTYH*, within the analytical scope of the original panel. These negative findings reduce the likelihood of several established hereditary CRC syndromes (3) but do not exclude pathogenic variant classes outside the validated detection range of the assay.

Family history revealed striking very-early-onset colorectal neoplasia. The proband’s elder sister (III-2) was diagnosed with rectal adenocarcinoma at 19 years of age in 2016. Histopathological examination of her surgical resection specimen demonstrated moderately differentiated rectal adenocarcinoma measuring approximately 3.5 × 3.0 × 0.8 cm, extending through the bowel wall into the perirectal adipose tissue. Metastatic carcinoma was identified in 1 of 14 examined regional lymph nodes, and the distal resection margin was negative for tumor. Immunohistochemical examination showed retained expression of MLH1, MSH2, and MSH6, weak retained PMS2 expression, and negative p53 staining. The proband’s father (II-2) underwent complete colonoscopy at 47 years of age in August 2022. Two rectal polyps measuring approximately 0.6 cm and 0.8 cm were identified and subsequently removed endoscopically. Histopathological examination of the submitted rectal polyp tissue demonstrated tubular adenoma. The paternal grandfather (I-2) was reported to have died from a rectal malignant tumor of unspecified histological type. No genetic testing or archival pathological material from the grandfather was available. Retained Sanger sequencing records were available for six individuals: the proband (III-3), her elder sister (III-2), her father (II-2), her brother-in-law (III-1), and her sister’s two children (IV-1 and IV-2). The heterozygous *SDHB* c.268C>T variant was detected in the proband, her sister, and her father, whereas III-1, IV-1, and IV-2 showed the wild-type sequence at this locus. Although this family demonstrates clustering of colorectal neoplasia and familial occurrence of the *SDHB* variant, the father’s tubular adenoma is not phenotypically equivalent to colorectal carcinoma, and the affected paternal grandfather was not genetically characterized. Therefore, the available data are insufficient to establish disease-specific co-segregation between the *SDHB* variant and colorectal cancer. The family pedigree and representative Sanger sequencing chromatograms are shown in **Figures 2A, B**, respectively.

**Figure 2:**
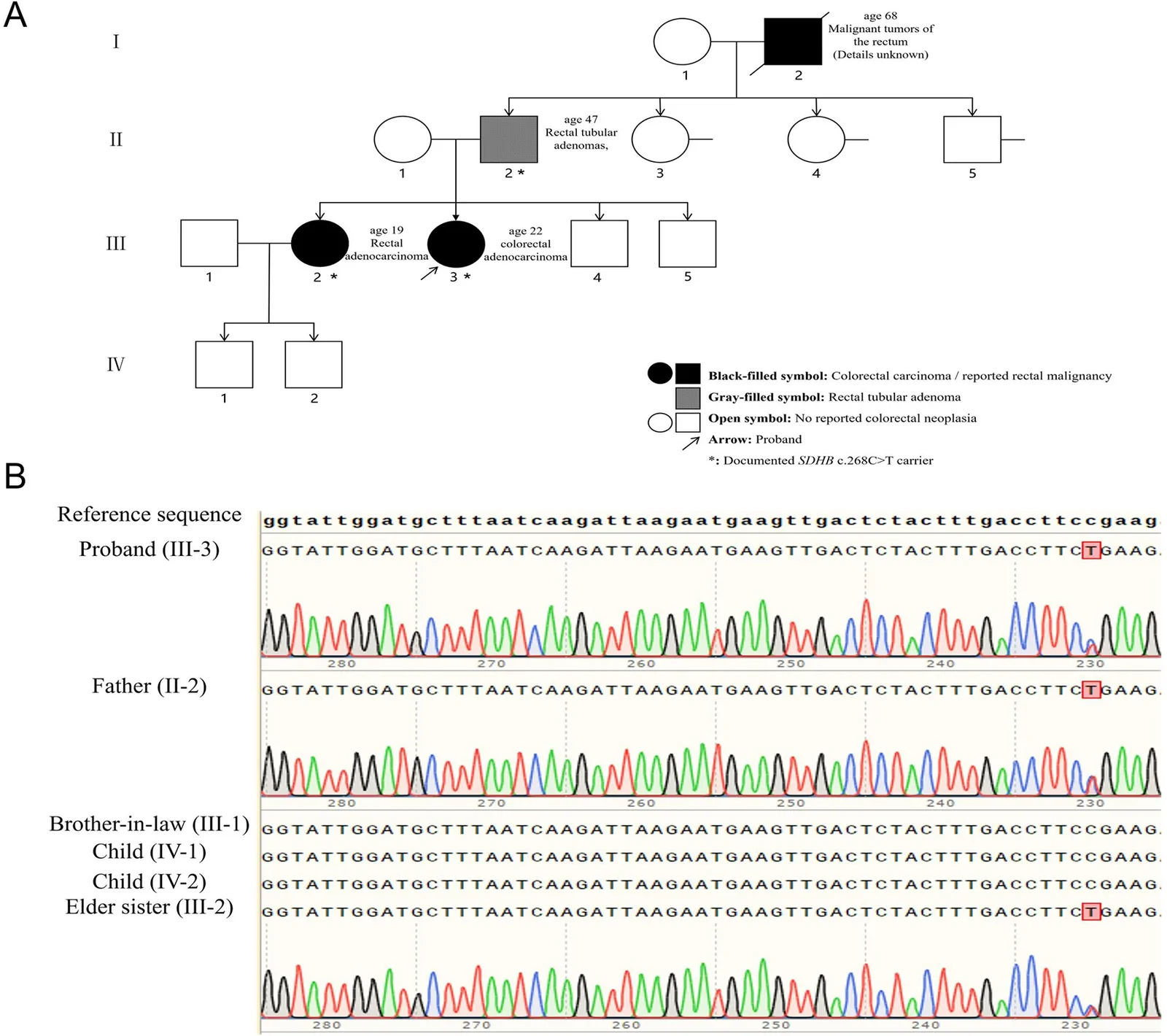
Family pedigree and Sanger sequencing of the familial *SDHB* c.268C>T (p.Arg90Ter) variant. **(A)** Pedigree of the family. The proband (III-3) was diagnosed with colorectal adenocarcinoma at 22 years of age, and her elder sister (III-2) was diagnosed with rectal adenocarcinoma at 19 years of age. Their father (II-2) had two rectal tubular adenomas at 47 years of age. The paternal grandfather (I-2) was reported to have a rectal malignant tumor of unspecified histological type. The arrow indicates the proband. Asterisks indicate individuals with documented carriage of the *SDHB* c.268C>T variant based on retained sequencing records. Solid symbols indicate colorectal carcinoma or reported rectal malignancy, the gray-filled symbol indicates rectal tubular adenoma, and open symbols indicate individuals without reported colorectal neoplasia. **(B)** Sanger sequencing chromatograms from the six individuals with retained sequencing records. The heterozygous *SDHB* c.268C>T variant was detected in the proband (III-3), her elder sister (III-2), and her father (II-2), whereas the proband’s brother-in-law (III-1) and her sister’s two children (IV-1 and IV-2) showed the wild-type sequence at this locus.

## 3 Discussion

Hereditary colorectal cancer encompasses a clinically and genetically heterogeneous group of disorders, including Lynch syndrome, familial adenomatous polyposis, *MUTYH*-associated polyposis, polymerase proofreading-associated polyposis, and other less common cancer-predisposition syndromes. Nevertheless, some patients with marked familial clustering or very-early-onset CRC remain genetically unexplained after standard hereditary cancer testing. Such families may reflect currently unrecognized genetic factors, variant classes not detected by the original assay, polygenic susceptibility, shared environmental influences, or combinations of these mechanisms. Comprehensive characterization of such families may therefore help identify clinically relevant hypotheses for future investigation.

The most striking clinical feature of this family is the occurrence of colorectal carcinoma in two sisters at exceptionally young ages: rectal adenocarcinoma at 19 years in the elder sister and colorectal adenocarcinoma at 22 years in the proband. Their father had two rectal tubular adenomas at 47 years of age. This pattern strongly raises concern for an underlying familial predisposition to colorectal neoplasia; however, the responsible genetic mechanism remains unresolved.

The *SDHB* c.268C>T (p.Arg90Ter) variant detected in this family is a known pathogenic loss-of-function variant in established *SDHB*-related tumor predisposition syndromes (5, 6). However, pathogenicity in these disorders does not establish a causal role in colorectal cancer. The available family data likewise do not demonstrate formal disease-specific co-segregation. Although both sisters with colorectal carcinoma and their father carry the variant, the father’s phenotype consisted of tubular adenoma rather than colorectal carcinoma, and the affected paternal grandfather was not genetically characterized. Tumor-level findings in the proband also provide limited support for an *SDHB*-driven mechanism. The *SDHB* variant showed a VAF of 20.75% in the tumor, no biallelic *SDHB* inactivation was demonstrated, and SDHB protein expression was retained by immunohistochemistry. Although retained SDHB expression does not necessarily establish normal SDH enzymatic function, interpretation of SDHB immunohistochemistry can involve diagnostic pitfalls; nevertheless, the present staining pattern does not provide conventional immunohistochemical evidence of an SDH-deficient tumor phenotype (8). Several non-mutually exclusive explanations should therefore be considered for the apparent colorectal-predominant phenotype. The germline *SDHB* variant may represent an incidental hereditary finding unrelated to the colorectal phenotype. Alternatively, incomplete penetrance and variable expressivity of *SDHB*-associated tumor predisposition may contribute to phenotypic heterogeneity (9), while unidentified hereditary factors or tissue-specific somatic events could also be involved. The somatic *TP53* frameshift alteration and *SMAD4* copy-number loss identified in the proband may contribute to colorectal tumor biology; however, functional cooperation between these alterations and germline *SDHB* has not been demonstrated.

Interestingly, p53 immunostaining was reported as negative in the colorectal tumors of both sisters. In the proband, tumor sequencing identified a somatic *TP53* frameshift alteration; however, corresponding tumor-sequencing data from the elder sister were unavailable. These observations do not establish a germline *TP53* syndrome but reinforce the need to consider alternative hereditary mechanisms that may not have been fully assessed by the original targeted assay. Overall, the present findings support the existence of a remarkable very-early-onset familial colorectal cancer phenotype but do not establish *SDHB* as its causal susceptibility gene.

From a clinical perspective, individuals carrying pathogenic *SDHB* variants should continue to receive surveillance for established *SDHB*-associated tumor risks according to current recommendations for hereditary *SDHx*-related tumor predisposition. Recommended follow-up includes annual clinical assessment, biochemical testing with plasma-free or urinary fractionated metanephrines every two years during childhood and annually in adults, and MRI-based imaging approximately every 2–3 years (10). In contrast, there is currently insufficient evidence to establish an *SDHB*-specific colorectal surveillance protocol. In this family, intensified colorectal surveillance should be justified primarily by the extraordinary family history of colorectal carcinoma at 19 and 22 years of age and by each individual’s colorectal findings, rather than by the *SDHB* genotype alone. Several limitations should be acknowledged. First, this report involves a single family, and only a subset of relatives has retained and independently verifiable genetic data; clinical and genetic characterization of several extended family members also remains incomplete, limiting formal disease-specific segregation analysis. Second, archival tumor material from the affected paternal grandfather was unavailable, and additional tumor-level molecular comparison among affected relatives was therefore limited. Third, although the *SDHB* p.Arg90Ter variant has established pathogenic significance in recognized *SDHB*-related disorders, the present study did not perform CRC-specific functional experiments, and no biallelic *SDHB* inactivation was demonstrated in the proband’s tumor. Thus, an *SDHB*-driven mechanism of colorectal tumorigenesis cannot be established. Finally, the prevalence and enrichment of pathogenic germline *SDHB* variants among patients with familial or very-early-onset CRC remain unknown, and comprehensive germline genomic analyses capable of assessing all copy-number and structural variants were not performed. Alternative hereditary mechanisms therefore cannot be completely excluded.

## 4 Patient perspective

Being diagnosed with colorectal cancer at a very young age, particularly in the context of my elder sister’s previous diagnosis of rectal cancer, made me concerned about whether cancer might run in my family. Genetic testing and counseling helped me better understand that a germline *SDHB* variant was identified in several family members, but that its relationship with colorectal cancer has not yet been definitively established. I understand the importance of continued postoperative follow-up and appropriate colorectal surveillance. I also hope that further investigation of my family may help clarify the hereditary basis of the disease and provide useful information for the health management of my relatives.

## 5 Conclusions

This report describes a remarkable family in which two sisters developed colorectal carcinoma at 19 and 22 years of age, respectively, while their father had rectal tubular adenoma. A known pathogenic germline *SDHB* c.268C>T (p.Arg90Ter) variant was identified in the two sisters and their father. However, the current family segregation data, tumor molecular findings, and immunohistochemical evidence are insufficient to establish *SDHB* as a hereditary colorectal cancer susceptibility gene. In particular, the proband’s tumor retained SDHB protein expression and did not demonstrate biallelic *SDHB* inactivation, while alternative hereditary mechanisms remain incompletely excluded. The present observation should therefore be considered hypothesis-generating. Additional independent families, larger cohorts of very-early-onset and familial CRC, comprehensive germline genomic analyses, and CRC-specific functional studies will be required to determine whether germline *SDHB* variants contribute to colorectal tumorigenesis.

## Data Availability

The original contributions presented in the study are included in the article/**Supplementary Material**. Further inquiries can be directed to the corresponding author.
