# Supplementary material for: Case Report: A germline SDHB c.268C>T (p.Arg90Ter) variant in a family with very-early-onset colorectal neoplasia
Source: Front Oncol. 2026 Oct 16;16:1938314. doi: 10.3389/fonc.2026.1938314 (PMC13719406; doi:10.3389/fonc.2026.1938314)
Supplement: Supplementary file 1 [file SupplementaryFile1.docx]

**Supplementary Methods Genetic Analysis**

**1. Panel Information and Methodology**

Germline variant analysis was performed using the "Yuan Shi Bo®" (源适博®) hereditary cancer screening panel provided by Genecast Biotechnology (Wuxi, China). This commercial NGS panel targets the coding exons and adjacent intronic regions (±20 bp) of 87 genes associated with hereditary cancer syndromes. The detection utilizes hybrid capture technology for whole-exome enrichment on a high-throughput sequencing platform (Illumina-based). The methodology has been validated according to Clinical Laboratory Improvement Amendments (CLIA) and College of American Pathologists (CAP) standards, and the assay is approved by the National Medical Products Administration (NMPA) as a Class III medical device (Registration No. 20213400151).

**2. Variant Interpretation**

Sequence variants were aligned to the human reference genome (GRCh37/hg19). Pathogenicity was classified according to the 2015 American College of Medical Genetics and Genomics (ACMG) and the Association for Molecular Pathology (AMP) standards and guidelines. The analyzed 87-gene list is provided in the table below:
